# A community-based program to reduce acute rheumatic fever and rheumatic heart disease in northern Australia

**DOI:** 10.1186/s12913-021-07159-9

**Published:** 2021-10-20

**Authors:** Vicki Kerrigan, Angela Kelly, Anne Marie Lee, Valerina Mungatopi, Alice G. Mitchell, Rosemary Wyber, Anna P. Ralph

**Affiliations:** 1 Menzies School of Health Research, Charles Darwin University, PO Box 41096, Casuarina, Northern Territory 0811 Australia; 2 George Institute for Global Health, Level 5, 1 King Street, Newtown, New South Wales 2042 Australia; 3 Telethon Kids Institute, 15 Hospital Avenue, Nedlands, Western Australia 6009 Australia; 4 Royal Darwin Hospital, Darwin, Northern Territory 0811 Australia

**Keywords:** Aboriginal health, Rheumatic heart disease, Community led healthcare, Health services, Social determinants of health

## Abstract

**Background:**

In Australia’s north, Aboriginal peoples live with world-high rates of rheumatic heart disease (RHD) and its precursor, acute rheumatic fever (ARF); driven by social and environmental determinants of health. We undertook a program of work to strengthen RHD primordial and primary prevention using a model addressing six domains: housing and environmental support, community awareness and empowerment, health literacy, health and education service integration, health navigation and health provider education. Our aim is to determine how the model was experienced by study participants.

**Methods:**

This is a two-year, outreach-to-household, pragmatic intervention implemented by Aboriginal Community Workers in three remote communities. The qualitative component was shaped by Participatory Action Research. Yarning sessions and semi-structured interviews were conducted with 14 individuals affected by, or working with, ARF/RHD. 31 project field reports were collated. We conducted a hybrid inductive-deductive thematic analysis guided by critical theory.

**Results:**

Aboriginal Community Workers were best placed to support two of the six domains: housing and environmental health support and health navigation. This was due to trusting relationships between ACWs and families and the authority attributed to ACWs through the project. ACWs improved health literacy and supported awareness and empowerment; but this was limited by disease complexities. Consequently, ACWs requested more training to address knowledge gaps and improve knowledge transfer to families. ACWs did not have skills to provide health professionals with education or ensure health and education services participated in ARF/RHD. Where knowledge gain among participant family members was apparent, motivation or structural capability to implement behaviour change was lacking in some domains, even though the model was intended to support structural changes through care navigation and housing fixes.

**Conclusions:**

This is the first multi-site effort in northern Australia to strengthen primordial and primary prevention of RHD. Community-led programs are central to the overarching strategy to eliminate RHD. Future implementation should support culturally safe relationships which build the social capital required to address social determinants of health and enable holistic ways to support sustainable individual and community-level actions. Government and services must collaborate with communities to address systemic, structural issues limiting the capacity of Aboriginal peoples to eliminate RHD.

## Introduction

In northern Australia, Aboriginal peoples suffer the highest documented burden of acute rheumatic fever (ARF) in the world with an age-standardised rate of initial ARF of 71.9 per 100,000 population among Indigenous Australians, compared with 0.60 per 100,000 for non-Indigenous Australians between 2015 and 2017 [1]. This largely preventable disease has been linked to disparities grounded in colonial practices which disadvantage Indigenous peoples through restricted access to culturally safe healthcare, housing and education [2–5]. Modelling shows that unless the structural, social and clinical determinants of health are addressed 2835 Indigenous Australians will develop RHD by 2031, of whom 1356 will develop severe RHD and 663 will die prematurely [6].

ARF and its complication, rheumatic heart disease (RHD), usually starts in childhood, stretches into adulthood and causes premature death. ARF is an autoimmune disease which develops as an abnormal response to an untreated group A streptococcus (Step A) infection of the throat or skin. Recurrent ARF, or severe initial ARF, leads to permanent heart valve damage called RHD [7]. Approaches to controlling the progression of ARF have focussed on delivery of secondary prophylaxis with painful monthly penicillin injections to affected individuals however new cases of ARF continue to develop, predominantly amongst children and adolescents.

As part of synthesising and piloting recommendations developing during the End Rheumatic Heart Disease Centre of Research Excellence (END RHD CRE) - an outreach-to-household pragmatic intervention for select Aboriginal communities in the Northern Territory (NT) of Australia was developed. The aim was to move beyond focusing on monthly penicillin injections as the mainstay of RHD management to address upstream determinants of health. In particular, to explore feasibility and acceptability of local approaches to support individuals and families affected by ARF/RHD who have limited access to healthcare, acceptable housing, and health information. A largely quantitative evaluation [8] of the project’s initial 12 months found through high retention rates that it appeared to be acceptable to the community and that biomedical approaches to disease management should be “augmented by outreach-based supports delivered by Aboriginal Community Workers in conjunction with community needs”. However further qualitative research was required, over a longer period, to better understand community experiences with the program and develop recommendations for iteration and development.

This paper expands on Wyber et al’s^8^ findings by providing qualitative evidence gathered over two years from the same study. Our aim is to explore how the outreach-to-household model was experienced by study participants: Aboriginal Community Workers (ACWs), affected ARF/RHD families and individuals, and healthcare providers in the three remote Aboriginal communities in the Northern Territory (NT). The goal is to identify priorities and possibilities for expansion which fulfill community expectations and needs.

## Methods

### Study design

This pragmatic intervention was a collaboration between NT communities, government run and Aboriginal community-controlled health clinics, the Department of Territory Families, Housing and Communities and clinician-researchers. The qualitative component draws on elements of Participatory Action Research (PAR). PAR has strong ties to bottom-up community led processes, facilitates the collective production of knowledge and encourages transformation through reoccurring cycles of action, research and reflection [9–11]. The overall project has been described elsewhere [8] however a summary is provided for context.

### Study setting

**Fig. 1:**
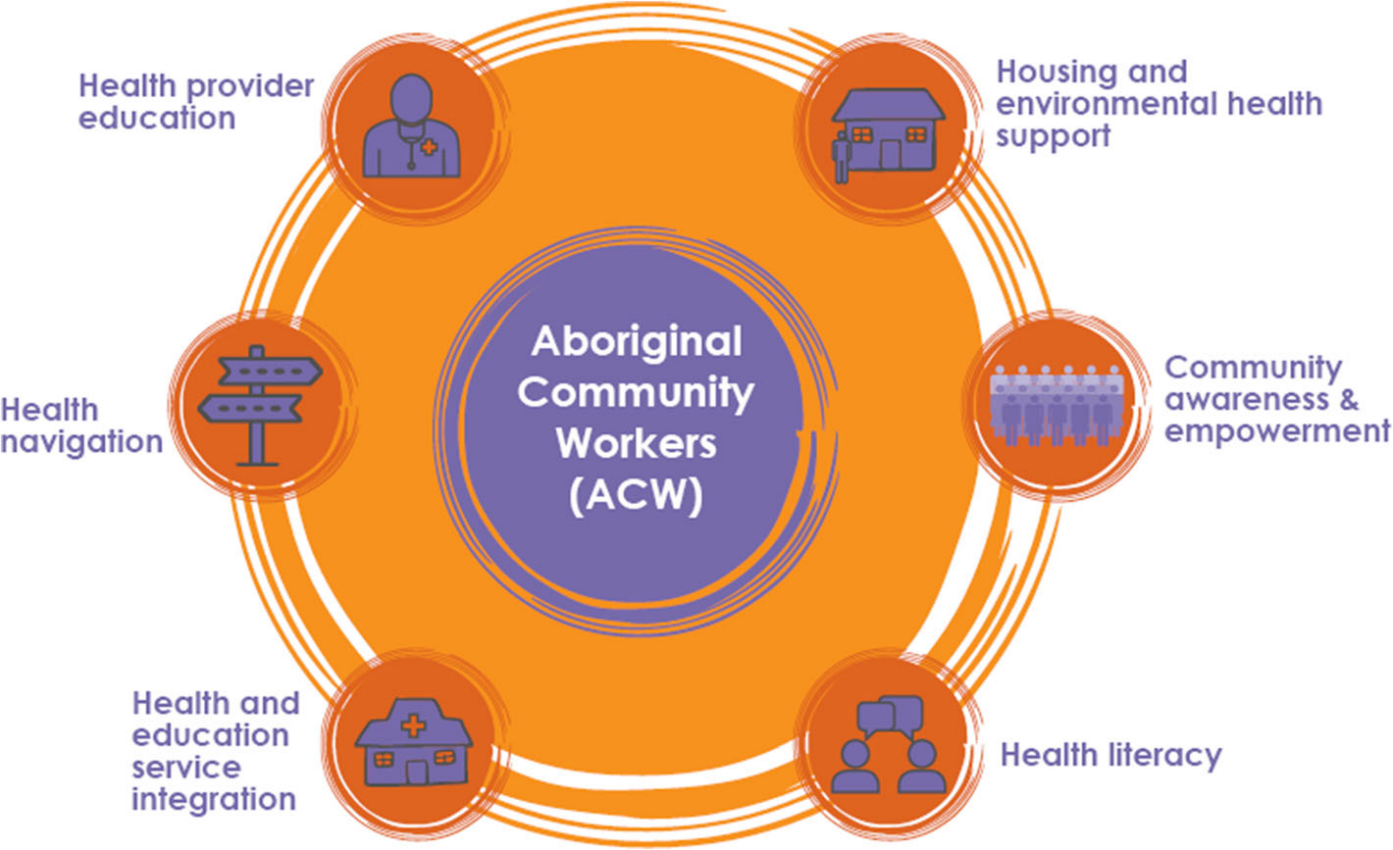
*Activity domains of the outreach-based support model*^8^

Three remote communities in the NT’s Top End, each with more than 90% of residents identifying as Aboriginal [12], agreed to participate and are referred to as sites A, B and C. Site A, population 401, is an island community accessible by a 30-min flight from Darwin. Site B, population 363, is accessible by road 400 km south east of Darwin. Site C, population 87, is 50 km from site B via an unsealed road which limits access during the monsoon season. ACWs were employed for 16 h per week: they received training in data collection and ARF/RHD to assist affected families to navigate their healthcare needs. The ACW’s central role is illustrated in Fig. 1. The Darwin based non-Aboriginal project manager supported ACWs with regular community visits (weekly/fortnightly/monthly depending on staff availability) and was available as required via telephone. Project staff community visits stopped between March 2020 and June 2020 due to COVID-19 travel restrictions.

### Participant sampling

ACW’s were employed as researchers and educators plus interviewed as research participants. Potential ACWs, who were respected within their communities and had experience as health professionals and/or community researchers, were identified through discussions with community leaders and clinic staff. As per PAR, participants are both the researcher and the researched; they have a vested interest in the topic and lived experience to share [10, 13]. A purposeful sampling strategy was used to identify families and clinic health professionals who could provide “information rich cases” [14].

### Data collection

 Interviews and field reports were collected. Consenting ACWs and family members participated in semi-structured individual or group interviews which were conducted as “yarning” [15] sessions. Yarning is an Aboriginal research method that creates a two-way learning opportunity in which ACWs, families and project staff shared stories and information about ARF/RHD. Individual clinic health professionals’ interviews explored the viability of the model according to existing health service practices. Discussions were conducted in English by non-Indigenous researchers (VK, AK, NF and AM) except one ACW interview which was undertaken with an Aboriginal language interpreter with VK. Interviews were recorded and transcribed verbatim except for two in which permission to record was declined and the researcher took notes. Project manager and project officer field reports provided context into the sociocultural and environmental determinants which impact health outcomes.

### Data analysis

Analysis draws on critical theory [16] which prioritises marginalised voices [17]. Aligning with PAR, critical theorists aim for structural social change to eliminate the systems which maintain Aboriginal health inequity. A hybrid inductive-deductive thematic analysis was conducted [18]. Initial inductive coding using NVIVO 12 was conducted separately by VK and AM. Codes were then deductively refined into themes already identified in Fig. 1: the domains of activity. Pseudonyms have been assigned to protect participant identity.

### Ethics

The Northern Territory Department of Health & Menzies School of Health Research Human Research Ethics Committee approved the research (HREC 2017–2898).

## Findings

Data was collected between March 2018 to June 2020. Fourteen participants (12 Aboriginal and two non-Aboriginal) took part in two group yarning sessions and 13 semi-structured interviews lasting between 9 min and 1 h and 13 min. Discussions with ACWs and families affected by ARF/RHD from each site occurred more than once: during the program’s early stages and after 18 months. Clinic health professionals were interviewed once at the end of the data collection period. Four ACWs, two adult males with a history of ARF, four mothers of children who had ARF and one grandmother of a child who had undergone open heart surgery for RHD, shared stories. Three clinic health professionals were interviewed: both non-Aboriginal clinic managers from site A and site B/C and a senior Aboriginal Health Practitioner (AHP) from site A. AHPs at site B/C were unable to be interviewed due to COVID-19 travel restrictions. Thirty-one project field reports were also analysed.

### Participants’ perspectives of local communities

As the program focused on addressing determinants of health, participants were asked to describe their communities. Field reports provided context regarding available services. ACW Sylvia, a Traditional Owner at the site A island community, described the beauty of her home:

*“It’s a beautiful place, based on the front beach … .The community actually sits, like in the middle of a valley, and you’ve got a hill over this side, and you’ve got a hill over that side, and the community’s in the middle.”* – Sylvia, ACW, site A

ACW Annie has lived at site B all her life and her family lives at site C. She says her community has active strong Aboriginal leaders:

*“If there’s an issue with something happened, the Elders and everyone, the strong leaders get together and they sort it out straight away the next day.”* – Annie, ACW, site B/C

Whilst both ACWs Sylvia and Annie were employed part time by the project, employment opportunities for participating families was scarce; only two had steady employment, and one worked two jobs. Other participating families regularly asked the project manager about work opportunities. Educational opportunities were also limited: many children left their homes to attend boarding school to complete high school. None of the communities have a police station so communities are required to develop their own systems to manage issues as noted in a field report: *Clinic bolted down yesterday due to someone with a metal bar outside – smashed tail lights of clinic vehicle.*

Site A is serviced by a government run clinic and at Sites B/C the health service is provided by an Aboriginal Community Controlled Health service. Both clinic managers reported children were healthier than previous generations although rates of chronic disease and illness is still unacceptably high. Three ARF/RHD affected participants were hospitalised during the study: two for ARF and one boy for RHD requiring cardiac surgery. ARF and RHD are only some of many health-related issues facing residents. Death due to accidents, chronic illness or suicide weighs heavily on every community.

Susan shared her family’s story: she has diabetes, her partner has lung problems, her daughter has had ARF, her son is medicated for behavioural issues, her youngest daughter has an “iron problem” and her two-year-old niece has kidney problems. Recently her children’s grandfather died. She is unable to find a job and her partner is also unemployed. There are six adults and three children living in their house. A further two adults have been visiting for 5 months: they drink beer and smoke marijuana. Concerned a crowded house and unhealthy behaviours will exacerbate her children’s illnesses, Susan has asked the visitors to move out however accommodation alternatives are limited. Susan’s story is not uncommon.

### Findings according to domains of activity

Findings are presented under seven headings in accordance with the six domains of activity and the central role of the ACW (Fig. 1).

### Aboriginal Community Workers

Three females were employed at site A and one female and one male at sites B/C. All spoke the language local to their communities. Twelve months into the project three ACWs had ceased employment. At the time of writing, Sylvia at site A and Annie at sites B/C had been employed for three years. ACWs who consented to interviews enjoyed the project work which they framed as ‘*helping people’*. Annie is a qualified, but not practicing Aboriginal Health Practitioner, and in addition to her work as an ACW held elected positions on council, housing, and health boards. She was motivated to do the ACW work out of concern for future generations:

*“I’m so passionate about the young ones that have rheumatic heart disease … I work very closely with the family because my great concern, they’re only young, got to make sure that they keep their heart healthy and grow up to really understand.”* – Annie, ACW, site B/C

Sylvia had previous experience as a community researcher. Her daughter received penicillin injections for possible ARF for one year. She explained ARF and RHD has affected her family:

*My sister-in-law got that too, my brother and his partner, so they both got that too … My brother had it when he was 5 years old and grow up, and it came back again too.* – Sylvia, ACW, site A

All participants were related within their community, either biologically or through the kinship system including the two non-Aboriginal long-term resident clinic managers who were ‘adopted’. A strength of the program’s design was that families trusted the ACWs to share information enabling information to be diffused quickly through networks:

*“ … when you share information to those family members, that family member will talk to another family member … Then they’ll know, ‘Oh yeah, that’s what Annie is doing’. So, a small community, you cannot keep anything quiet. Everything just goes like bushfire … it’s a really good thing for me”.* – Annie, ACW, site B/C

Relationships come with expectations, obligations, risks and rules. One female ACW explained, following cultural protocols, it was inappropriate to talk to some men she was related to. Another ACW spoke of a previous experience (unconnected to the project) in which health staff were wrongly blamed by the community for the death of a family member who was undergoing ARF treatment. As a result, the ACW feared physical retribution known as payback. The incident eventually strengthened the ACW’s resolve to improve their community’s understanding of ARF and RHD. Despite the associated pressures all participants agreed the project’s success relied on local Aboriginal community members implementing the project. Site A’s non-Aboriginal clinic manager Paul said: “*You don’t want a whitefella telling someone else what to do.”* Site B/C’s ACW Annie explained the logic of having local workers:

*“Indigenous people working on the ground from that community, it works well. It doesn't work if an outsider thinks that they can come in. To me, it won't work like that. You got to get local people on the ground to do the job. You can work beside that person, we can show you, we can help you. But you always got to remember Indigenous people have the first preference and they are the one that knows the family, whose got the problem and it works well.”* – Annie, ACW, site B/C

ACW’s ability to fulfil the job description was impeded by the need to travel to larger centres to personally access health and social services. Project officer reports from a 5-month period in 2019 showed that one ACW was available to work alongside the project officer during site visits just 50% of the time. The stressors experienced by families (described above) also exist for the ACWs who generally worked as solo operators whilst juggling family, cultural and professional responsibilities. Families and ACWs agreed more ACWs should be employed to ensure the support promised by the project can be consistently delivered and so work can expand to include all families in the community, not just those affected by ARF. A project officer report from site A also noted when ACWs work in a team, job satisfaction and productivity improved. ACWs requested more group training to reduce the isolation of being solo workers, create team building opportunities and improve knowledge.

*“You need to get more workers based in this community to give out that good information about how can we stop rheumatic heart from blowing up.”* – Annie, ACW, site B/C

The cornerstone of the ACW’s work was a regular household survey (results published in Wyber at al [8]). The survey had a dual purpose to gather data about health hardware, presence of potential Strep A infections, number of household occupants and maintenance issues and to stimulate educational conversations around ARF prevention. At the start of the study, family participant Polly (mother of a child with ARF/RHD) said contact with the ACW reminded her to clean her bathroom and report any housing problems however as the project progressed, she reported feeling bored by the repetitive survey questions. Similarly, the plan to visit clients in their homes to complete surveys was not acceptable to many participants. Collecting data from homes was difficult at site A where ACW’s described entering someone’s home under those circumstances as *“embarrassing*”. Responding to community preferences, site A ACWs set up outside the local shop with educational material to show family and friends. The visibility of the ACWs in their branded research institute t-shirts meant education was available to all:

Julia, ACW site A*: “Some people are interested to come and look, they come and join us and we talk to them.* (We say) *‘You’re welcome to read, what the heart does to you’, ya know, ‘heart problem’.”*

Sylvia, ACW site A*: “*(I say to them) *‘We’re trying to help you mob’.”*

Project officer reports revealed that visibility of ACWs in the community, and subsequent contact, acted as a reminder for individuals to receive their monthly penicillin injection.

### Community awareness and empowerment

Educational resources supplied by the umbrella organisation RHD-Australia and the project team were provided to improve community awareness and empower ACWs and families. Pamphlets, laminated images showing skin sores and heart valves (healthy and damaged), heart models and electronic tablets loaded with videos were used by the project team to educate ACWs. Subsequently the ACWs could use the resources to educate families. Despite the supposed availability of resources families reported a lack of awareness about the cause of ARF. Chloe’s 10-year-old daughter diagnosed during the project said: “*There’s no information … I don’t know how it happened or what to do.”* At site A, Bill who was diagnosed 10 years previously as a teenager, said no one had explained to him how he can prevent his two children from developing ARF. When probed further he recalled watching a video about ARF/RHD but was unable to remember the details.

Bill captured the overwhelming attitude to preferred modes of education: primarily he wanted to have a conversation with a trusted source. He did not want pamphlets. Susan from site A said she wanted to “*stretch*” the conversation with people like ACW Sylvia with whom she felt *“comfortable*”. The same message came from site B families who requested a community information session hosted by the trusted ACW with invited health specialists. One family actively sought information independently. Mum Polly at site A searched the phrase “rheumatic heart” on YouTube to find videos to show her 9-year-old son who had been diagnosed with ARF two years previously. She said extra phone credit would be appreciated so she can continue to access the internet to educate her family. The benefits of their knowledge was observed by researcher VK in 2020. The boy returned from playing with his friends. He immediately washed his hands with soap. Asked to explain his behaviour he said: *“Because germs might go inside me and I might get sick”.* In contrast to Bill’s disinterest in written materials, Polly also said she appreciated an educational book she received when her son was originally hospitalised with ARF:

*“Because me and my partner usually, well, when [son] first got that rheumatic heart fever, me and my partner had to go through that book five, six, well, you can say 20 times, just to read over and over what's good and best for him.”* – Polly, mother, site A

There was limited evidence to support the idea that the wider community, outside of participating families, benefited from the project. Bill revealed he doesn’t talk to his family about ARF by explaining: *“They just always busy that mob.”* Although at Site A clinic manager Paul believed community awareness and empowerment occurred through multigenerational living which created opportunities for knowledge transfer amongst families:

*“I think it’s a generational change. You won’t see change in the short term but in the long term, I think it will be quite beneficial. –* Paul, clinic manager, site A

Attempting to raise community awareness at site B, primary school children were supported to write and perform a song about ARF symptoms and management. An accompanying video clip featuring local school children, some with ARF, was also produced. Parents said the video was very popular and shared amongst their children: *“Replay, replay, replay all day.”-* Lara, mother, site B. The song was also performed by school children at the annual community arts, culture and sports festival which attracts thousands of visitors annually.

To expand community awareness and empower families with knowledge, the ACWs said their groundwork should be supported by an education campaign broadcast through local media and published in community newsletters. Whilst English is commonly read in communities, it is not commonly spoken. To that end ACWs requested educational videos and radio segments in local languages recognising that children predominantly communicate in the language they speak at home.

### Health literacy

Data collection focussed on health literacy related to ARF/RHD not general health literacy. Knowledge of causation, symptoms, treatment and prevention was limited at the start of the project. Findings are described as per ACW and family knowledge.

**ACWs:** ACW health literacy improved across the life of the project. In an initial workshop, site A’s Julia asked pertinent questions: “*What do* [heart] *valves do?*” and “*What causes joint pain?”.* Annie explained, despite prior training as an AHP, her knowledge of ARF/RHD improved during the project:

*“But, when I did that research, I said, ‘Oh, so, skin sores and sore throat, it makes your heart not good’. Before when I was an Aboriginal Health Practitioner, we all thought skin sores only damage your kidneys …… I swear, that’s what I was trained when I was an Aboriginal Health Practitioner.”* – Annie, ACW, site B/C

Knowledge of germ theory varied. Site A’s Cressida had recently completed school where she learnt basic germ theory. As understanding developed due to training, ACWs described germs as something akin to small worms or insects invisible to the naked eye. Annie believed people need to understand germ theory, but education needs to be delivered in a comprehensible way using visual aids and in local languages. During an interview, Sylvia used a small heart model to explain the impact the strep germ can have on the heart:

*“The strep germ makes your heart sick. Makes your heart valve not pump and your blood not pump in your valve. It makes it bad inside.” –* Sylvia, ACW, site A

ACWs Annie and Sylvia correctly explained contemporary medical understandings of ARF causation (strep germ transmitted by open wound, coughing, touching), symptoms of ARF precursors and ARF (skin sores, sore throat and sore joints, fever), treatment (attend clinic) and prevention (penicillin needle, personal hygiene). Some of the ACW’s understandings of ARF causation were inaccurately broad (acquisition through scratching or lack of household cleanliness).

Increased ACW knowledge around skin sores resulted in at least one person at site A receiving much needed medical attention. Sylvia was concerned an elderly woman with a skin sores was showing signs of ARF. She reported her concerns to the clinic manager. The elderly woman was flown to Royal Darwin Hospital where the infection, unrelated to ARF, was treated.

After two years of project activities neither Sylvia nor Annie could confidently explain the distinction between ARF (a self-limiting acute set of symptoms) and RHD (the chronic form of heart valve damage which may be asymptomatic). Sylvia said: “*the two are the same, they come all together.”* Annie said ACWs want a more comprehensive understanding of ARF/RHD treatment which could be satisfied by observing health professionals working with patients at Royal Darwin Hospital.

**Participant families:** The time since diagnosis of ARF/RHD among families participating in interviews and yarning circles ranged from six weeks to 16 years. Knowledge of ARF/RHD was not associated with the time since diagnosis. Jackson at site C has been receiving penicillin injections for 16 years since the age of 5 yet appeared to have no knowledge of RHD causation. He also said he had never heard of the strep germ. Bill was diagnosed over 10 years ago and incorrectly believed ARF is hereditary:

*“Because what I was told is it runs through the family. Like my dad’s brother had it before and then my big sister got it and myself.”* – Bill, adult male, site A

Susan also worried ARF was hereditary. She asked if her 15-year-old daughter would pass ARF on to her children: “*If she has a partner and they have a baby, the baby carries that as well?”.* Susan’s daughter had been receiving penicillin injections to control ARF for 3 years. Susan believed that ARF could be prevented through a healthy diet:

“*The only thing I can see is if you eat the right food: fish, kangaroo, turtle, and vegies, yoghurt, fruit. Not much drink – soft drink just give it like 2 weeks and then they can have a snack, little snack if they went without it for 2 weeks”*. – Susan, mother, site A

Knowledge of RHD causation for Susan, Bill and Polly’s families who were receiving regular penicillin injections to prevent ARF and engaging in hygiene practices, did not improve during the project. In both baseline and final interviews, Polly said playing in the dirt and the rain, muddy puddles, with dogs and in swimming pools can cause ARF:

*“I really don't know but I can say that he may have got it from swimming in a dirty pool with other kids. Yeah well, he didn't have many sores, so yeah, I can just blame the pool.”* – Polly, mother, site A

Polly’s lack of knowledge meant she worried her daughter will contract ARF from playing on her son’s bed or her son will have a recurrent ARF episode. This was despite her vigilance regarding her son’s personal hygiene as reported above plus washing her son’s clothes and bedding separately. Many parents were worried about sharing kitchenware, beds and towels. At the end of the study period, all families remained concerned about transmission of ARF and RHD. There were mistaken beliefs that illness could be transmitted by household members with RHD or who had had ARF, in the absence of active Strep A infection.

Considering the monthly recommendation to attend a clinic to receive a painful injection to control ARF progression, it was concerning that most families were unable to elucidate the purpose of the injection. Polly was the only participant who correctly explained her son received injections to “*kill germs*”. This was explained despite Polly not articulating the connection between germs and ARF causation.

Families who experienced the most severe ARF/RHD complications appeared to have better health literacy than other families. Grandmother Kimberley escorted her 9-year-old grandson 3500 km south to a major centre for open heart surgery. She had comprehensive knowledge of causation, symptoms, prevention and treatment. She was the only person who named the strep germ as the cause of ARF/RHD.

#### Housing and environmental health support

Plumbing and water problems were common across all sites. At site B/C, ACW Chloe said it was normal not to have hot water and Annie said, *“One young fella didn’t have hot water for three years”.* At site A, mum Susan explained she had to constantly “*boil a jug*” to provide warm water to clean her son’s skin sores. A project manager report from site C documented serious infrastructure issues:

“*verandah* (sic) *filling up with water, not draining. Part of toilet wall missing. Can smell sewage”.* – Field report, site C

To improve housing and environmental health support, the Department of Territory Families, Housing and Communities was a project stakeholder. This meant the ACW’s authority was recognised by the department, which resulted in improving the response time of housing repairs in some cases to a few days. At site A, Bill’s lounge room flooded every wet season for 5 years. Concerned for his children’s safety, he reported the problem to the relevant government housing department but Bill reported “*they said they had no money.”* The problem was reported to ACW Sylvia and the issues were fixed.

*“ … because when they asked by themselves it takes three or four months, so health like me, I work for health, I got a report, and come straight away, like the next day.”* – Sylvia, ACW, site A

At site B, Jackson said the home he shared with his father and grandfather had *“no hot water, windows broken, doors need fixing, and kitchen. My dad is sick and he is on medication*”. ACW Annie helped Jackson’s family move into temporary housing for one month while repairs were completed. However, a short time after moving back home, the water problem reoccurred.

Three contributing factors to maintenance delays were identified. Families were not aware of their rights. At Site A, Susan believed she had no authority to report problems to the Department of Territory Families, Housing and Communities because she was not the leaseholder and her daughter with ARF (who’s diagnosis could elevate the priority of health hardware maintenance for that household) was away at boarding school. At her home, water was leaking through a ceiling fan and she feared her kids would get an electric shock. She added: “*When we had big rain, I nearly slipped and broke my leg.”* At site A, reporting of housing issues was sometimes hampered by the department’s office opening hours (weekday mornings only). At site B it was easier as the ACW was a member of the local housing reference group and was also a relative of the Housing department officer. Finally, project manager reports revealed maintenance delays were further compounded by confusion over who was responsible for housing. At site B/C most homes were owned and maintained by the Department of Housing and others by a non-government organisation. For example, at site C the Department did not own a condemned home but the tenant, a person living with ARF client, understood they lived in a Department managed property.

Household overcrowding and mattress sharing was difficult to address. An original idea in the study design was to promote ‘strep free zones’ by asking families to prioritise children with a prior ARF or RHD diagnosis for single bedrooms, or their own mattress if possible. This was deemed impractical. Site B/C’s ACW Annie reported that grandmother Kimberley and her grandson who underwent open heart surgery were living in a three-bedroom home with 16 others. The boy slept on his own mattress in the lounge room with his grandparents.

*“It is impossible to think of asking them to put one person with RHD in one room. There are not enough rooms and too many needs”* – Annie, ACW, site B/C

Attempting to address the issue, Annie advocated for four residents to move into one of three new homes under construction. But asking families to live in separate houses was difficult. Reportedly Kimberley did not wish to move to a less crowded home due to fond family memories at the current location. Annie said building more houses was a short-term fix and the issues which lead to overcrowding need to be addressed:

*“But I always don’t think about the overcrowding … Look behind, the other problems, other issues in Indigenous communities.”* – Annie, ACW, site B/C

#### Health provider education

ACWs did not have the capacity or authority to provide ARF/RHD education for health professionals. Site A and B/C clinics were managed by long term (20 years+) non-Aboriginal staff who were qualified nurses, John and Paul respectively. There were four AHPs at site A and five AHPs at site B/C. Site B/C clinic manager John said ARF and RHD was not a major concern in the community instead he named diabetes as the community’s biggest health issue. From site B/C during the study period, a 9-year-old boy underwent cardiac surgery, and a 26-year-old male was hospitalised. New ARF cases continued to be diagnosed. An 8-year-old child who lived in a well-maintained home, had not reported skin sores nor complained of a sore throat, was diagnosed with ARF. Additionally, at site B, Chloe’s child was initially misdiagnosed after presenting to the clinic with a sore knee. Chloe was encouraged by mum Lara (her daughter has ARF), to take her child back to the clinic because ‘*it could be rheumatic’*. Chloe’s child was then diagnosed with ARF. The interaction, occurred several months into the project after Lara had received education form ACW Annie, illustrates an instance of good community knowledge of symptoms, but poor recognition of potentially significant symptoms by clinic staff.

Poor recognition of ARF/RHD symptoms may be attributable to the high turnover of health professionals in remote clinics, often on short term contracts, and fly in fly out weekly doctor visits. Staff turnover can result in distrust in the health service and fractious community relations because transient staff are unaware of community needs and cultural protocols. At site B/C Annie preferred to work with Aboriginal Health Practitioners saying she doesn’t want to *“humbug*” (harass) nurses or doctors, who turnover rapidly:

*“I am trying to work closely with* [senior AHP]. *Trying to identify one staff member to connect with. Mununga* (white people) *always come and go.”* – Annie, ACW, site B/C

Recognising these issues and the complexities involved with diagnosis, both clinic managers requested staff training. Site A had four people newly diagnosed with in the last year, the clinic manager would like to see zero new diagnoses:

*“Well, I’d like to see no rheumatic fever in the community because you’ve got to get a needle every month and that’s the biggest deterrent. Yeah, and you don’t see it in mainstream. You only see it out here, so we’ve got to be able to get rid of it, knock it on the head. There are complications and major complications if they’re untreated or misdiagnosed.”* – Paul, clinic manager, site A

Despite the aim of the project to go beyond the provision of monthly penicillin to focus on primordial and primary prevention, clinic health professionals believed the ACW’s main role was to ensure people get their scheduled injection (secondary prevention):

*“I think sort of Annie’s main job is to help remind us to make sure we're chasing them, and people know that they're due, get them in and get them done.” –* John, clinic manager, site B/C

#### Health navigation

Most families requested ACW assistance to navigate services except for Polly who reported being well supported by her partner and mother-in-law. The ACWs worked with families to navigate Department of Housing issues (detailed above) and the clinic. However, ACWs reported their capacity was limited by project resources. ACWs requested a car to complete their work in the allotted 16 h per week to improve efficiency and avoid aggressive (“*cheeky*”) dogs. ACW Annie explained she worked unpaid overtime because her ability to travel 50 km between site B and C is reliant on clinic staff.

No major issues were reported regarding acceptability of clinic service. Some participants were clinic employees: Bill was employed as the site A clinic driver and site B/C’s ACW Annie was the chair of the local Aboriginal Community Controlled services’ health board. Families had relationships with AHPs at both clinics. At site A clinic manager Paul has lived in the community for 20 years: “*I’ve been here a fair few years now, so they know I don’t growl too much”.* Site B clinic manager, John, believed because of a long running health promotion initiative between the school and the clinic, kids are “*used to coming to the clinic and it’s not nasty”*.

Despite reported good relationships, engaging with the clinic was at times hampered by other health and family issues. Adherence to secondary prophylaxis monthly injections for people with ARF or RHD remained problematic. At the time of interview, the clinic managers reported at site B two people were overdue for their prescribed secondary prophylaxis dose and at site A four children were overdue. At site A, interviewer (AK) remarked that people seemed sad and inquired if that might be the reason why four children had not attended the clinic for their needles. The site A senior AHP, Nancy, replied there had recently been three deaths in the community which affects people’s ability to attend the clinic because multi-day ceremonies are often conducted on the deceased’s traditional land away from the community.

Both ACW Annie and Sylvia incorporated the task of reminding family when they were due for their needle into their roles. Annie was concerned the clinic did not do enough to ensure injections were delivered on time so she would remind individuals about the importance of receiving their prescribed injections when due. Site A’s ACW Sylvia successfully encouraged a boy, who was overdue for his needle, to attend the clinic. Sitting under a shady tree, less than 200 m from the clinic, she showed mother and son pictures of healthy and damaged heart valves. Sylvia shared the conversation:

“[I said] *‘We don’t want* [your child] *to have that bad valve … He might go to Darwin or Adelaide, get operation, get sick. Show* [your son] *that.’ So she went, ‘Okay,* [son] *come here.* [Son] *if you don’t get that needle, you’ll have this.’ He looked in there, ‘That valve in your heart, bad one and if you get needle all the time you get good valve, it’ll be better,’ she said to him … * [he said] *‘Okay then Mum, what you waiting for? We got to go now, got to go get that needle now’.* ”- Sylvia, ACW site A

#### Health and education service integration

A lack of formal arrangements between the research team and service providers meant ACWs were unable to sustain activity in this vital domain.

Clinic professionals requested more information from the research team and increased engagement with the ACW including attendance at clinic staff meetings to share research findings and provide information on ARF/RHD. The site A clinic manager expressed a desire to collaborate with the research team to address housing issues. Clinic managers believe the ACW role could potentially compliment the roles of AHP and nurses who have a heavy workload. Nancy, a senior AHP at site A, valued the ACW’s ability to support families and answer questions:

*“Makes my job a bit easier, yeah whereas we just jab them and then we just leave them and sometimes talk to Sylvia.”-* Nancy, AHP, site A

Engagement with schools was inconsistent. At site B ACW Annie attended the primary school to visit four children with ARF and their classmates to talk about prevention strategies. This occurred fortnightly for 12 months until problems arose between school staff and the community. At site A school visits were less frequent however when ACWs attended they reported a handwashing exercise with glitter potion and UV light to demonstrate effectiveness of handwashing was an engaging and powerful educational tool:

*“So they like that thing now with the germs … .they keep going outside trying to scrub their hand really hard, but ‘No, it’s still in there now (laughs)’.* [They ask] *‘How come they’re still here and I wash my hands?’. ‘You don’t wash it properly,’ I said.”* – Sylvia, ACW, site A

One family reported a serious communication issue with their daughter’s interstate boarding school. Susan’s daughter was hospitalised during the school term but Susan was unaware. Susan said to the school:

*“‘Every time you take* [my daughter] *for appointment or the doctor we want to see her about the result, you make sure you tell me and my partner, because we’re her parents’ … If it’s like serious or something, she would be really scared to tell me. She’s far away from us, and it’s very sad for us, you know. She told me when she came back for bush holiday last year, and I don’t really know much about that, so that probably broke my heart too.”* - Susan, mother, site A

Project officer reports showed there were opportunities to engage with non-health hegemonic services. At site A, the training and employment centre, arts, men’s and early childhood and family centre expressed interest in expanding their scope of workshops to include ARF/RHD education. At site A, Sylvia sporadically attended the employment and training centre to talk with women about ARF/RHD. At site B, Annie was unable to arrange approval to attend the government run training centre because the centre was without a coordinator when the project activities commenced. Collaborating with established organisations to expand services may improve relations with one local land council who, according to field reports, was generally dissatisfied with research projects overall.

## Discussion

Qualitative findings from this pragmatic intervention provide insights into how the model could be redeveloped to better align with community needs and values. We found the current model in which the ACW was expected to execute all aspects of project implementation was not achievable. ACWs addressed two of the six domains of activity (Fig. 1): housing and environmental health support and health navigation. To a much lesser degree, ACWs also supported community awareness and empowerment and improved health literacy. ACWs did not have the capacity to provide health professionals with education or ensure health and education service integration. When considering service redesign, the lived experience of individuals “within the context of the constraints and possibilities for individual agency“” [2, 19] must be considered. To that end, we assert the ACWs’ inability to address each domain was restricted by service provider issues and the project design. The domains of activity (Fig. 1) were originally derived from synthesising research findings and collaboration with Aboriginal health leaders experienced in ARF/RHD prevention [6]. Whilst the model centred community leadership the project was not genuinely community led because leaders from participating sites did not contribute to project design. We believe the model has the potential to be used in other communities affected by RHD, if the program of work is led by the community and aligns with community priorities and values. Transplanting this program requires site specific tailoring, which includes building trusting relationships and “structural changes to mainstream and government funded services” [20]

Future implementation and potential expansion to additional sites should focus on supporting strong relationships and creating systemic and structural change through strategic collaborations. These areas will be discussed below.

Firstly, the most successful components of the project are largely attributable to strong interpersonal relationships between key individuals. The Darwin based project team built respectful relationships with the two ACWs who remained employed for three years. Consequently, the ACWs established themselves as experts on ARF/RHD in their communities. Participating families trusted the ACWs because of their homophilous (shared beliefs, values and culture) relationship [21] which reduced the risk of families feeling like they were monitored by an authority [2]. Aboriginal peoples have lived lives under “extraordinary surveillance” [22]. This impacts on how people interact with mainstream services. Healthcare interactions for most Aboriginal peoples are shaped by experiences of powerlessness and racism [23, 24] however the power imbalance that commonly occurs between hegemonic service providers and local people was reduced by the ACW. In this balanced relationship with the ACW, people living with ARF could exercise autonomy which led to improved engagement with health services. An example is provided in the conversation reported between an ACW, a mother and child which resulted in the boy choosing to attend the clinic to receive his overdue penicillin injection.

To build on the foundational work which has occurred, the ACW’s position should be strengthened. ACWs articulated their preference to collaborate more closely with each other and the project team. Employing more ACWs, particularly men so gender roles can be respected, is required. ACWs and AHPs reported productive working relationships and clinic managers also valued the ACWs contribution. In remote clinics of the size participating in this study, doctors visit once a week, nurses move around on short term contracts and specialist services are provided increasingly via telehealth [25]. Even if clinic staff are stable there is a perception that non-Indigenous staff are not trustworthy [23, 26]. Embedding ACWs in the local clinic may go some way to addressing these issues.

Trustworthy relationships between ACWs and families meant knowledge of ARF/RHD improved although, recognising the complexity of the diseases, families requested more information from trusted individuals in their preferred language. Knowledge gaps were evident regarding causation, transmission of infection and prevention. All families remained anxious that ARF was hereditary or could be transmitted through social activities (such as swimming pools). While the likelihood of developing ARF (and then RHD) does cluster in families due to genetic traits, those genetic traits are only weakly associated with ARF/RHD likelihood [27]. The far more important reason that ARF/RHD clusters in families is due to shared environmental risk factors (e.g. access to culturally safe healthcare and housing). It is imperative community members know ARF/RHD is preventable, not genetically inevitable.

Research globally has found limited access to health information and medical mistrust leads to late diagnosis and suboptimal management of infectious diseases [28]. While ACW and family relationships were a strength, the possibility of trust being eroded due to health professional knowledge gaps, or perceived knowledge gaps, are real. Our findings revealed one case of initial ARF misdiagnosis by clinic staff at site B/C. This has the potential to diminish the trust community members have in clinic staff. The site B/C clinic manager stated ARF/RHD is not a major concern but at least one child out of a population of 300 had open heart surgery during the study period and now has a shortened life expectancy. The clinic manager’s appraisal likely reflects the very high burden of other major health threats facing the community which swamp the reality of ARF rates being amongst the highest internationally. It also remains concerning that at the end of the data collection period, neither ACW could explain the difference between ARF and RHD. Considering research-based knowledge of ARF/RHD continues to evolve and recognising their own knowledge gaps, ACWs requested more group training opportunities.

Whilst our findings appeared to reveal gaps in knowledge among ARF affected families, that does not necessarily mean knowledge of ARF/RHD did not improve during the project. Researchers with a biomedical lens often assume Aboriginal peoples have misunderstood clinical health messages because when the message is retold it has been indigenised and is no longer recognisable compared to the biomedical worldview [29]. For example Polly did not articulate ARF is caused by the strep germ but she clearly stated the purpose of penicillin was to kill germs. When assessing knowledge transfer researchers also need to be mindful that in some Aboriginal cultures, discussing ill health may be construed as a threat because “predicting illness can imply involvement with sorcery to cause the illness” [30]. This may be one of the reasons why Bill had not spoken to his family about ARF. Furthermore, an individual’s capacity to retain, and retell, information may be restricted by competing priorities relating to housing, employment concerns and food security. A 2019 health survey found 43% of Indigenous people in remote communities had gone without food in the previous 12 months [31]. Haynes et al [2] argue that health messages which privilege biomedical knowledge support the colonisation of Indigenous healthcare. To that end, we recognise that asking Aboriginal peoples to explain ARF/RHD in the same manner it was explained may be another form of colonisation.

We also recognise the biomedical model of passing information from expert to patient, used in this project, may not be the most effective way to teach in this context. Kelly and Barker [32] argue the expert to patient dissemination model is only effective for patients with acute conditions. This is supported by the extensive knowledge demonstrated by grandmother Kimberley who was able to explain in detail her grandson’s condition after he underwent open heart surgery. However, the model of education from expert to patient does very little to affect behaviour change amongst those who feel they are managing their chronic conditions [33]. The belief that giving people information will affect behaviour is “wrong and unscientific. Giving people information does not make them change.” [32] All people, including families like Kimberley’s who did not want to move out of their overcrowded home, make choices which may not benefit their physical health when the choices on offer do not align with their values or life circumstances [32]. Therefore, future implementation of this program requires greater emphasis on structural changes which can support individual behaviour change.

Individual behaviour change requires support in the form of better access to resources and services [32, 34]. This was appreciated when the study model was devised however more intersectionality is needed, emphasising the cross-departmental linkages and large-scale investment needed to ensure individuals and families can live healthy lives. This includes improving the quality of housing which the Australian government recognises impacts on education, employment and health engagement [35]. Housing authorities need to address inadequate infrastructure which negatively impacts on the social capital [36]. An estimated 37% of Indigenous Australians live in a home with major structural problems [37] and least 40% of houses in remote NT Aboriginal communities are overcrowded [38]. Crowding is a leading cause of high rates of infection with Strep A, the trigger for ARF [39]. In a landmark case the NT Supreme Court found renters in one NT community should be compensated for the standard of housing which was deemed inhumane and uninhabitable [40]. Across most remote Aboriginal communities, including sites A, B and C renting from the housing department is the only option which means families have no control over housing infrastructure. Whilst project activities successfully assisted some families repair housing issues faster than usual, the families themselves remained disempowered and disengaged from housing services. Housing authorities should consider how to better engage with communities to ensure public housing tenants better understand their rights and responsibilities [41].

Other structural changes to consider could include altering microenvironments [33, 42] which support sustainable behaviour change without stigmatising individuals. This has previously successfully occurred in remote Queensland Aboriginal communities by replacing soft drinks with water in shop fridges resulting in a reduction in the consumption of sugary drinks [42]. In this context, nudge [43] approaches to reducing Strep A skin infection could include distribution of hygiene consumables such as soap for washing hands and bodies. To be effective, microenvironment changes must be supported by changes to the macro environment which could include educational initiatives in schools and across media that promote hand washing and changes to community infrastructure which supports incidental washing, such as water parks, swimming pools or community ablutions blocks [6]. To avoid another paternalistic policy, this nudge model requires full community co-design and support [42].

Finally, strategic collaborations with education and employment services and community organisations should be explored whilst continuing the ACWs refined role. Minority groups exposed to unequal treatment in housing, medical care and employment experience higher rates of infectious disease [44]. To address the determinants of health, the scope of services involved should expand to include employment and training centres, family, childcare and community services and men’s groups. As ARF incidence peaks in 5–14 year old children [7], formal arrangements with schools, both local and boarding schools, are required to embed education into school curriculum. Engaging with established arts centres and sports clubs in communities should be considered. Community football clubs have been found to be culturally safe welcoming spaces which have the potential to positively impact on health outcomes at a community level [45]. Social marketing campaigns, designed and delivered by local leaders, delivered through Aboriginal and mainstream media outlets should be considered [46]. Previous health warning campaigns have triggered / generated resistance rather than compliance amongst Aboriginal peoples as they are perceived to form ‘part of a broader apparatus of oppression’. [47] Therefore, health campaigns must be co-designed and delivered by local leaders in local languages [48, 49]. Collaborations such as these address the expressed wishes of both ACWs and clinic health professionals who believe it is important to share ARF/RHD prevention messages beyond affected families.

### Limitations

ACWs and other participants may have felt pressured to talk positively about the project fearing critical feedback could result in termination of employment of themselves or other project staff. This risk was mitigated by interviewers explaining the importance of critical feedback so project activities can expand according to community needs. At Site A, attempts were made to conduct patient interviews with local language interpreters however interpreters were not available. To ensure findings presented did not “whitewash” Aboriginal perspectives, ACWs reviewed analysis and findings through individual and group discussions. As per PAR [11], ACW feedback was incorporated and ACW’s named as co-authors. Finally, we recognise that Aboriginal communities are not culturally homogenous, and these findings are relevant to three Top End, NT communities. However, there is a shared history of colonisation which has been well documented as a driver of poor health outcomes for Aboriginal peoples [50, 51] and we believe the experiences documented here have relevance to similar jurisdictions with high rates or ARF/RHD.

## Conclusion

This study is the first sustained, multi-site effort to strengthen primordial and primary prevention of RHD in northern Australia. Community-led RHD prevention is a core component of the overarching strategy needed to eliminate RHD as a public health problem [6]. Our analysis identified strengths but also important limitations of this project. While the model was based on broadly stated Aboriginal community priorities and desired ways of doing, we recommend co-designing such programs with communities in the first instance, as a way of strengthening the ACW role. ACWs were placed at the centre of the study model, yet the small number of ACWs employed per community placed excessive onus for project delivery on too few individuals. Next steps will focus on three areas: expanding the skilled ACW workforce, ensuring knowledge of ARF/RHD improves amongst ACWs and health providers and finally, we assert it is paramount that government and community service providers engage more closely with communities at a local level to redesign systems and services to address health inequity.

## Data Availability

Data collected and analysed during the current study are not publicly available due to privacy issues and ethical considerations. Data may be available from the corresponding author on reasonable request.

## Abbreviations

ACW: Aboriginal Community Workers
ARF: Acute Rheumatic Fever
NT: Northern Territory
PAR: Participatory Action Research
RHD: Rheumatic Heart Disease
